# Upregulation of skeletal muscle PGC-1α through the elevation of cyclic AMP levels by Cyanidin-3-glucoside enhances exercise performance

**DOI:** 10.1038/srep44799

**Published:** 2017-03-20

**Authors:** Toshiya Matsukawa, Hideko Motojima, Yuki Sato, Shinya Takahashi, Myra O. Villareal, Hiroko Isoda

**Affiliations:** 1 Graduate School of Life and Environmental Sciences, University of Tsukuba, Tsukuba City, Ibaraki 305-8572, Japan; 2 Alliance for Research on North Africa (ARENA), University of Tsukuba, Tsukuba City, Ibaraki 305-8572, Japan; 3 Faculty of Life and Environmental Sciences, University of Tsukuba, Tsukuba City, Ibaraki 305-8572, Japan

## Abstract

Regular exercise and physical training enhance physiological capacity and improve metabolic diseases. Skeletal muscles require peroxisome proliferator-activated receptor-gamma coactivator-1α (PGC-1α) in the process of their adaptation to exercise owing to PGC-1α’s ability to regulate mitochondrial biogenesis, angiogenesis, and oxidative metabolism. Cyanidin-3-glucoside (Cy3G) is a natural polyphenol and a nutraceutical factor, which has several beneficial effects on human health. Here, the effect of Cy3G on exercise performance and the underlying mechanisms involved were investigated. ICR mice were given Cy3G (1 mg/kg, orally) everyday and made to perform weight-loaded swimming exercise for 15 days. The endurance of mice orally administered with Cy3G was improved, enabling them to swim longer (time) and while the levels of exercise-induced lactate and fatigue markers (urea nitrogen, creatinine and total ketone bodies) were reduced. Additionally, the expression of lactate metabolism-related genes (*lactate dehydrogenase B* and *monocarboxylate transporter 1*) in gastrocnemius and biceps femoris muscles was increased in response to Cy3G-induced PGC-1α upregulation. *In vitro*, using C2C12 myotubes, Cy3G-induced elevation of intracellular cyclic AMP levels increased *PGC-1α* expression via the Ca^2+^/calmodulin-dependent protein kinase kinase pathway. This study demonstrates that Cy3G enhances exercise performance by activating lactate metabolism through skeletal muscle PGC-1α upregulation.

Regular exercise and physical training enhance an athlete’s endurance in sports and prevent as well as alleviate numerous diseases, such as obesity, type 2 diabetes, sarcopenia and cardiovascular disease^1^. Physical activity promotes mitochondrial biogenesis and oxidative activity, which has a positive effect on an individual’s overall energy balance and age-related degeneration^2^. The expected lifespan without long-standing illness has been observed to be 8–10 years longer in physically active people than those who are not^3^. In the human body, the skeletal muscles are considered as the main organ of metabolism since they contain a large number of mitochondria^1^,^4^. The major positive impact of exercise is believed to be as a result from the activation and improvement of the function of skeletal muscle^1^,^2^. Regular exercise and physical activity are effective for enhancing endurance during exercise performance and provide overall benefit to human health^1^,^2^.

Peroxisome proliferator-activated receptor-gamma coactivator-1α (PGC-1α) is a transcriptional coactivator that plays an important role in the regulation of metabolism^5^. Exercise training has been linked to increase in skeletal muscle PGC-1α, attenuating several adaptations to exercise such as mitochondrial biogenesis, angiogenesis, oxidative metabolism activation, and muscle growth^1^,^2^,^6^. Enhancing muscle function and metabolism by exercise training contribute to the prevention and amelioration of obesity and metabolic disease^1^. Muscle-specific overexpression of PGC-1α in mice has been reported to increase exercise capacity, fatigue resistance, and oxygen uptake^7^. Furthermore, muscle-specific PGC-1α knockout mice are characterized by reduced exercise capacity, muscle function, oxidative metabolism activity, and abnormal glucose homeostasis^8^. This highlights the importance of PGC-1α as a regulator of exercise adaptation in skeletal muscle and the prevention of metabolic disease^1^,^8^,^9^.

Nutraceuticals, such as dietary fiber, probiotics, vitamins and polyphenols, have recently gained attention as alternative medicine because of the physiological benefits gained following their intake that help in the prevention of diseases^10^. For example, food supplement containing resveratrol, a polyphenolic compound, can attenuate exercise performance by activating the metabolic activity and mitochondrial biogenesis in skeletal muscles through PGC-1α expression^11^. Cyanidin-3-glucoside (Cy3G) is a polyphenol compound that is present in numerous colourful fruits and vegetables, such as black soybean, blueberry and grape^12^, has many positive health properties. Animal and *in vitro* model studies have shown that Cy3G has anti-obesity^13^ and anti-diabetic effects^13^,^14^. Additionally, the supplementation of red grape leaf extract containing Cy3G has been reported to enhance exercise capacity by activating fatty acid oxidation^15^. Furthermore, in human clinical trial done by Mazza G *et al*.^16^ reported that the consumption of blueberry powder containing Cy3G reduces high-fat meal-induced blood oxidation. However, despite the many health benefits that consumption of foods rich in Cy3G has been reported, the mechanism of its effect on exercise and muscle metabolism has not yet been elucidated. In this study, we investigated the effects and mechanism of how Cy3G regulate exercise and muscle metabolism using exercised mice and cultured skeletal muscle cells.

## Results

### Regulation of blood lactate and glucose levels enhanced exercise performance in Cy3G-administraed mice

**Figure 1:**
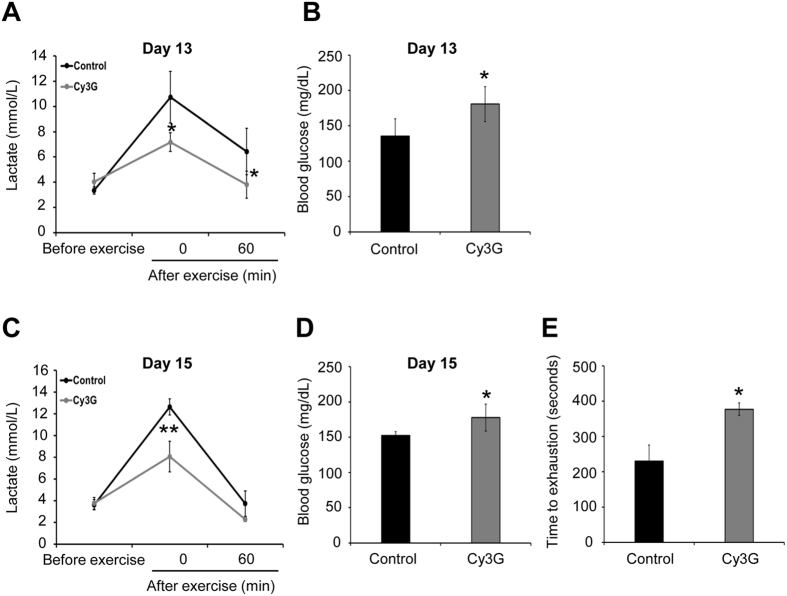
Regulation of blood lactate and glucose levels enhanced swimming time. Mice were trained to perform a swimming exercise and performed the exercise every other day for 14 days, and a swimming-until-exhaustion test was carried out on day 15. On days 13 and 15, (**A**,**C**) blood lactate levels before and after exercise (0 and 60 min) and (**B**,**D**) blood glucose levels after exercise (0 min) were evaluated. (**E**) On day 15, swimming time to exhaustion was measured. Values are expressed as the mean ± standard deviation. *P ≤ 0.05 and **P ≤ 0.01 indicate a significant difference from the control group.

Blood lactate levels during exercise are associated with the ratio between lactate production and clearance^17^,^18^. Therefore, blood lactate levels are strongly linked to exercise performance. To examine the effect of Cy3G on exercise performance (swim until exhaustion with a load corresponding to 10% of their body weight), mice were given Cy3G or water (control) orally for 15 days and were made to perform exercise (Supplementary Fig. S1). Blood lactate levels were measured on day 13 (10 min swimming) and day 15 (swimming until exhaustion) before and after the swimming exercise. Results showed that before performing exercise, the blood lactate levels were not statistically different between the control group and Cy3G-treated group (Fig. 1A and C). However, after exercise, as shown in Fig. 1A and C, the elevation of lactate was significantly lower in the Cy3G group on Day 13 (10.7 mM vs. 7.2 mM, *P* = 0.02) and Day 15 (12.6 mM vs. 8.0 mM, *P* < 0.01) compared to the control group. Additionally, the Cy3G group’s high glucose levels were maintained after exercise on Day 13 (136 mg/dL vs. 181 mg/dl, *P* = 0.02) and Day 15 (152 mg/dL vs. 178 mg/dl, *P* = 0.01) (Fig. 1B and D). Furthermore, as shown in Fig. 1E, Cy3G-administered mice did not get exhausted as fast as the control group as shown by the increased time to exhaustion (230 s  vs. 377 s, *P* = 0.02). The blood levels of urea nitrogen, creatinine, and total ketone bodies were then evaluated since these parameters are normally increased after exercise^19^,^20^. Cy3G-administered mice had lower levels of these biochemical components compared to control (Table 1: Urea nitrogen: 28.4 mg/dL vs. 22.2 mg/dL, *P* = 0.05, Creatinine: 0.20 mg/dL vs. 0.12 mg/dL, *P* = 0.02, Total ketone bodies: 1282.0 μM vs. 847.8 μM, *P* ≤ 0.01). Moreover, Cy3G-administered mice had increased gastrocnemius weight (0.23 g vs. 0.29 g, *P* = 0.04) and protein content (91.2 μg/mg vs. 96.9 μg/mg, *P* < 0.01) compared to control (Table 2 and Supplementary Table S1). The animals in three groups consumed on average the same amount of food (Table 2).

### Gastrocnemius and biceps femoris muscles lactate metabolism was enhanced in response to Cy3G-induced PGC-1α upregulation

**Figure 2:**
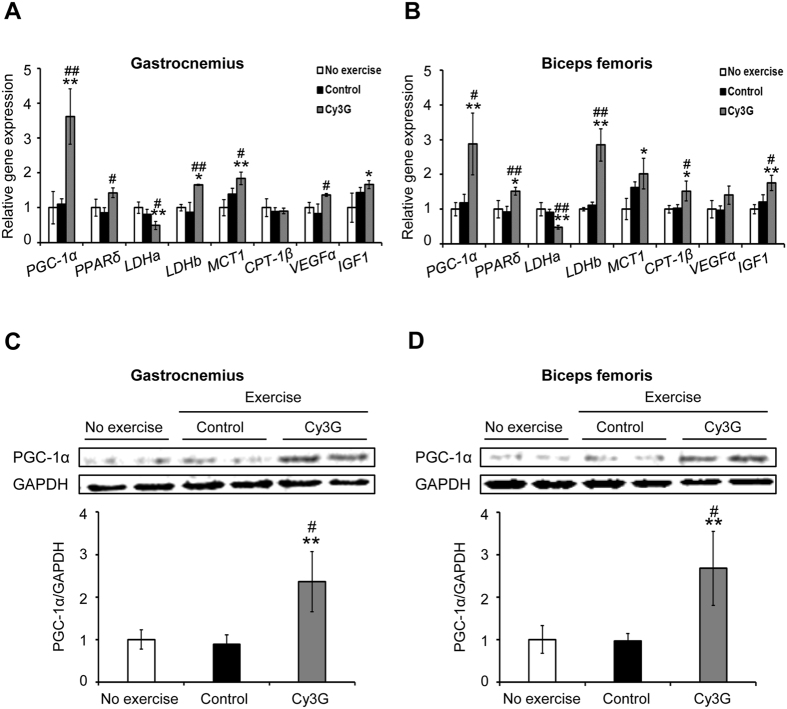
Lactate metabolism-related genes expression in the gastrocnemius and biceps femoris were controlled in response to Cyanidin-3-glucoside (Cy3G)-induced PGC-1α upregulation. Expression levels of PGC-1α and PGC-1α-targeted genes in the gastrocnemius (**A**,**C**) and biceps femoris (**B**,**D**) were evaluated. (**A**,**B**) Expression levels of mRNA were normalised to the *β-actin* expression level. (**C**,**D**) Protein expression levels were normalised to the expression of GAPDH. All gels were run under the same experimental conditions and the representative blots were shown. Values are expressed as the mean ± standard deviation and relative to the no exercise group. *P ≤ 0.05 and **P ≤ 0.01 indicate a significant difference from the no exercise group. ^#^*P* ≤ 0.05 and ^##^*P* ≤ 0.01 indicate significant difference from the control group.

Skeletal muscles are the major site of lactate production and clearance. Lactate produced by working muscle is released into the blood and taken up by muscle tissue as energy fuel^17^,^21^. To evaluate the effect of Cy3G on muscles, we collected the gastrocnemius and biceps femoris muscles, which are extensively used during swimming^22^. In both muscles, Cy3G administration greatly increased the expression of *PGC-1α* by 3.3 - fold (*P* ≤ 0.01) and 2.4 - fold (*P* = 0.02), respectively, compared to control group (Fig. 2A and B). Cy3G also increased the protein expression level of PGC-1α in gastrocnemius (Fig. 2C, 2.6 - fold, *P* = 0.02) and biceps femoris (Fig. 2D, 2.8 - fold, *P* = 0.02) compared to control group. PGC-1α is the transcriptional coactivator of several genes associated with adaptation to exercise such as lactate metabolism, free fatty acid (FFA) metabolism, angiogenesis (vascular endothelial growth factor α; VEGFα) and muscle growth (insulin-like growth factor 1; IGF1)^1^,^6^. PGC-1α in the skeletal muscle regulates blood lactate levels by promoting lactate dehydrogenase (LDH) B and monocarboxylate transporter 1 (MCT1) and decreasing LDH A expression^21^. LDH B catalyses the conversion of lactate to pyruvate and MCT1 facilitates lactate uptake^21^. Cy3G-administered mice had increased *LDH B* and *MCT1* expression in gastrocnemius (Fig. 2A, 1.9 fold, *P* = 0.01 and 1.3 - fold, *P* = 0.02) and biceps femoris (Fig. 2B, 2.5 - fold, *P* < 0.01 and 1.3 - fold, *P* = 0.13) compared to control group. LDH A catalyses the conversion of pyruvate to lactate^21^. *LDH A* expression was decreased by Cy3G in gastrocnemius (0.6 fold, *P* = 0.02) and biceps femoris (0.5 - fold, *P* ≤ 0.01) compared to the control group (Fig. 2A and B).

### Skeletal muscle cells lactate metabolism was enhanced in response to Cy3G

**Figure 3:**
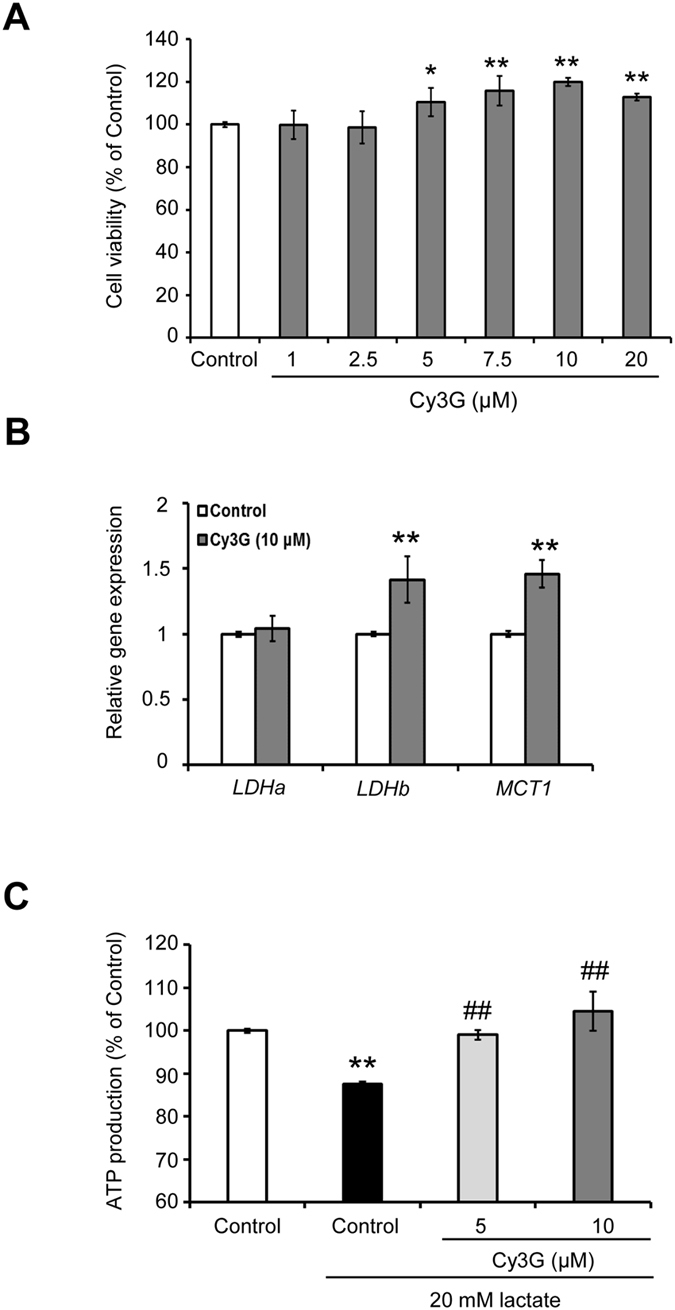
Lactate metabolism of C2C12 myotubes was enhanced in response to Cyanidin-3-glucoside (Cy3G). Differentiated C2C12 myotubes were treated with or without Cy3G for 24 h. After that, cell viability (**A**) and mRNA expression levels of *LDHa, LDHb* and *MCT1* (**B**) were evaluated. (**B**) Gene expression levels were normalised to the *β-actin* expression level. (**C**) After Cy3G treatment (24 h), C2C12 myotubes were cultured with serum- and glucose-free DMEM containing 20 mM lactate for 15 min. Intracellular ATP production in C2C12 myotubes was then evaluated. Values are expressed as the mean ± standard deviation of triplicate experiments. **P ≤ 0.01 indicates a significant difference from the control group. ^#^*P* ≤ 0.05 and ^##^*P* ≤ 0.01 indicate a significant difference from the lactate-treated control group.

Pharmacokinetic studies have revealed that after ingestion, Cy3G can be detected in its intact form in the blood^23^,^24^. To investigate the effect of Cy3G on lactate metabolism in skeletal muscle cells, we used C2C12 myotubes as a cellular model of the skeletal muscle. The effective and non-cytotoxic concentration of Cy3G was first determined using the 3-(4,5-cimethylthiazol-2-yl)-2,5-diphenyl tetrazolium bromide (MTT) assay. Treatment with Cy3G at 5 to 10 μM significantly increased the value of absorbance to 115% and 120%, respectively (Fig. 3A, *P* ≤ 0.01). Based on this result, we selected 10 μM as the Cy3G concentration to use in further experiments. Cy3G (10 μM) increased the *LDH B* and *MCT1* expression in C2C12 myotubes by 1.4- and 1.5-fold, respectively (Fig. 3B, *P* < 0.01). In humans, blood lactate levels are elevated to around 20 mM after a vigorous exercise, as what has been reported in other studies^25^,^26^. To evaluate the effect of Cy3G on lactate metabolism in skeletal muscle cells, Cy3G-treated C2C12 myotubes were cultured in serum- and glucose-free DMEM containing 20 mM lactate. As shown in Fig. 3C, Cy3G increased the intracellular adenosine triphosphate (ATP) production in comparison to the lactate-treated control (87% vs 104%, *P* < 0.01).

### Skeletal muscle cells mitochondrial content was increased by Cy3G-induced *PGC-1α* upregulation

**Figure 4:**
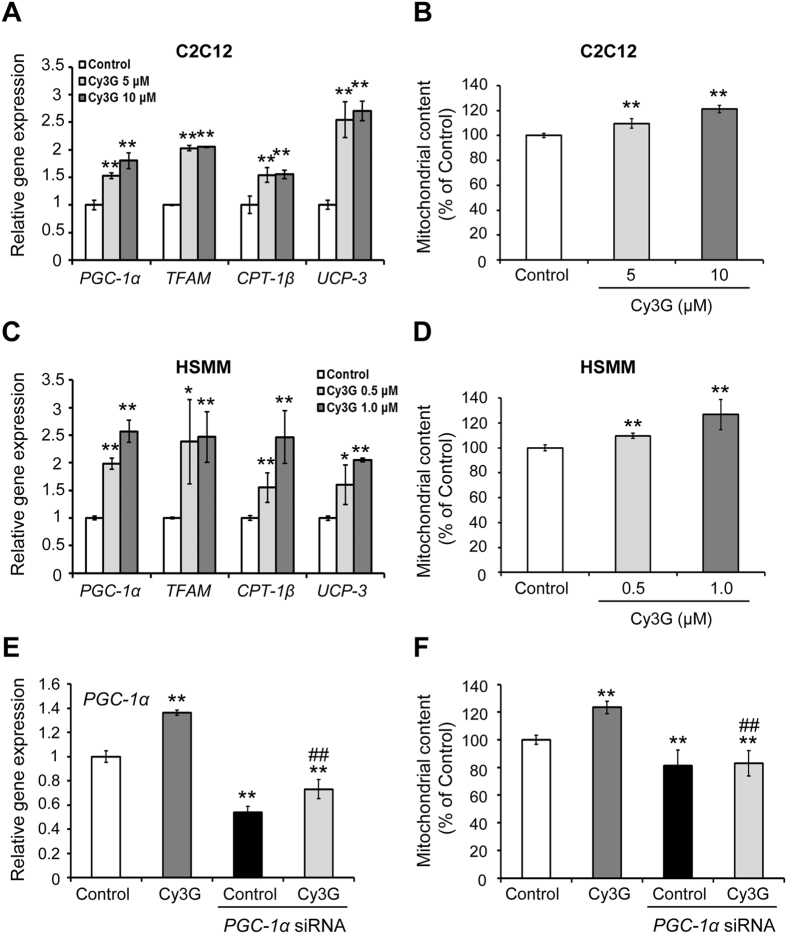
Mitochondrial content of skeletal muscle cells was increased by Cyanidin-3-glucoside (Cy3G)-induced *PGC-1α* upregulation. Differentiated C2C12 myotubes and HSMM were treated with or without Cy3G for 6 h (**A**,**C**) or 24 h (**B**,**D**), after which, the gene expression of *PGC-1α, TFAM, CPT-1β*, and *UCP-3* (**A**,**C**) and the mitochondria content (**B**,**D**) were evaluated. Differentiated C2C12 myotubes were transfected with *PGC-1α* siRNA or Control siRNA for 48 h and then, treated with or without Cy3G for (**E**) 6 h or (**F**) 24 h. Following treatment, *PGC-1α* mRNA levels (**E**) and mitochondria content (**F**) were evaluated. (**A**,**C**,**E**) Expression levels of mRNA were normalised to the *β-actin* expression level and expressed relative to the control. Values are expressed as the mean ± standard deviation of triplicate experiments. *P ≤ 0.05 and **P ≤ 0.01 indicate a significant difference from the control group. ^##^*P* ≤ 0.01 indicates a significant difference from the Cy3G group.

The mitochondria contain high level of LDH and MCT1, the expression of which is regulated by the PGC-1α^23^,^27^. PGC-1α regulates not only lactate metabolism but also mitochondrial biogenesis and functions^21^,^28^. To explore how Cy3G affects skeletal muscle cells mitochondria, the mitochondrial content and the mRNA levels of the genes involved in mitochondrial biogenesis and function were evaluated. Cy3G significantly increased the expression of *PGC-1α*, mitochondrial transcriptional factor A (*TFAM*) and the mitochondrial genes (*CPT-1β* and *UCP-3*) in C2C12 myotubes and human skeletal muscle myotubes (HSMM) (Fig. 4A and C). In addition, Cy3G significantly increased the number of mitochondria as shown by the increased in rhodamine 123 stain that was retained in the cells (Fig. 4B and D). However, the observed Cy3G-induced elevation of rhodamine 123 content was suppressed by *PGC-1α* knockdown (Fig. 4E and F).

### Activation of CaMKK–AMPK pathway through elevation of intracellular cAMP levels is involved in Cy3G-induced *PGC-1α* upregulation

**Figure 5:**
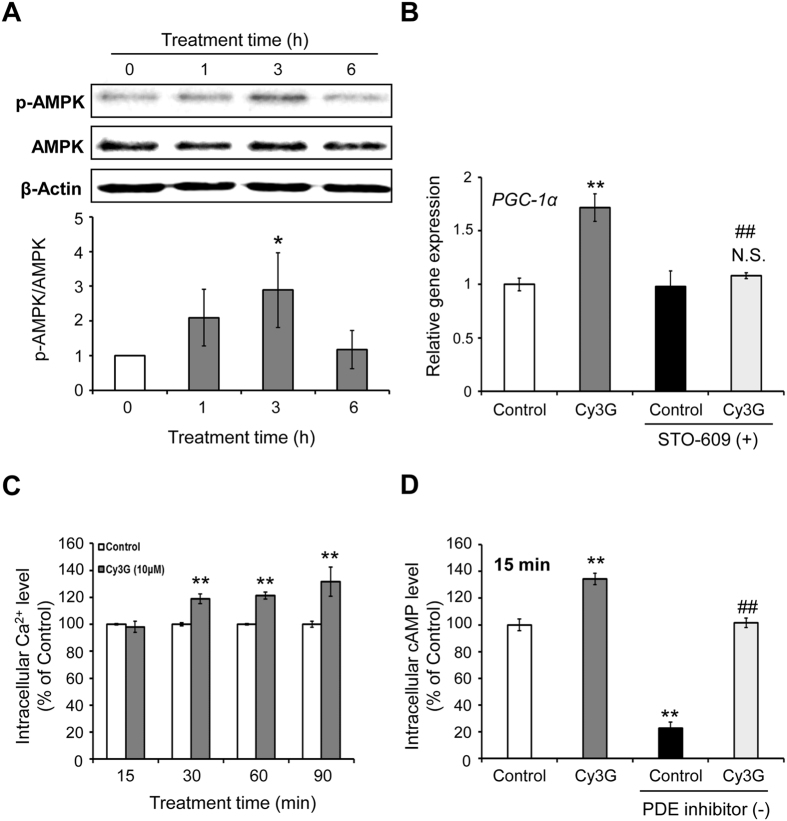
CaMKK–AMPK pathway and intracellular cAMP levels were involved in Cyanidin-3-glucoside (Cy3G)-induced *PGC-1α* upregulation. C2C12 myotubes were treated with or without Cy3G (10 μM) for 0–6 h and then phosphorylated AMPK levels were evaluated and values were normalised to the β-actin expression level. All gels were run under the same experimental conditions and the representative blots were shown (**A**). C2C12 myotubes were pre-treated with or without STO-609 (1 μg/ml) for 30 min. Cy3G (10 μM) treatment was then carried out with or without STO-609 for 6 h. *PGC-1α* mRNA levels were evaluated and values were normalised to the *β-actin* expression level (**B**). C2C12 myotubes were pre-incubated with Fluo4 AM for 30 min and subsequently treated with or without Cy3G for 15–90 min (**C**). C2C12 myotubes were treated with or without Cy3G (10 μM) and with or without PDE inhibitors (500 μM IBMX and 100 μM Ro20-1724) for 15 min. The intracellular cAMP level was then measured (**D**). Values are expressed as the mean ± standard deviation of triplicate experiments. *P ≤ 0.05 and **P ≤ 0.01 indicate a significant difference from the control group. ^##^*P* ≤ 0.01 indicates a significant difference from the Cy3G group. N.S. indicates that the mean value is not significantly different from that of the STO-609-treated control group.

The adenosine monophosphate (AMP)-activated protein kinase (AMPK), one of the main cellular energy sensors, is activated in response to a variety of stimulation such as cold temperature, exercise, hypoxia, and AICAR^29^,^30^. The activation of AMPK pathway promotes mitochondrial biogenesis through PGC-1α^29^. In this study, Cy3G treatment for 3 hours significantly increased the phosphorylation of AMPK by 2.88-fold (Fig. 5A, *P* = 0.03). Ca^2+^/calmodulin-dependent protein kinase kinase (CaMKK) and liver kinase B1 are kinases upstream of AMPK that induce AMPK phosphorylation^30^. However, *PGC-1α* upregulation by Cy3G treatment is inhibited when the cells were treated with STO-609, a CaMKK inhibitor^31^ (Fig. 5B). Cy3G increased intracellular Ca^2+^ and cyclic AMP (cAMP) levels (Fig. 5C and D). Elevated intracellular cAMP levels induce the release of Ca^2+^ from the sarcoplasmic reticulum^32^. As shown in Fig. 5C and D, 15 min after Cy3G treatment, an elevation of cAMP levels was detected but no change in the Ca^2+^ levels was observed. However, after 15 more min or 30 mins after treatment, an increase in Ca^2+^ level was detected.

## Discussion

The major finding of the current study is PGC-1α upregulation in skeletal muscle Cy3G enhances exercise performance. PGC-1α is the transcriptional coactivator of genes associated with adaptation to exercise such as lactate and FFA metabolism, angiogenesis, and mitochondrial biogenesis^1^,^6^. In obese individuals, PGC-1α significantly low, as what has also been observed in diabetes and cardiomyopathy patients^5^. Our study suggests that the increase of PGC-1α expression induced by Cy3G is useful in the management of obesity and metabolic diseases as well as in the enhancement of exercise performance.

Lactate is an anaerobic metabolite produced when glucose or glycogen is used as a fuel source. It produces H^+^ ions, which depresses muscle function and performance, and its accumulation in the muscles causes fatigue^18^. Additionally, hypoglycemia or decreased blood glucose levels during exercise can also cause fatigue, leading to exercise cessation^33^. *In vivo,* Cy3G group lowered the blood lactate level but increased the glucose level immediately after exercise (Fig. 1). The decrease in *LDH A* expression in Cy3G group suggests a decrease in lactate production and glucose metabolism. Elevation of skeletal muscle PGC-1α promotes lactate clearance ability^1^,^21^. It has been reported that exercise increases PGC-1α mRNA level but returns back to its “before exercise level” during the rest period such as 24 h after exercise^34^,^35^,^36^. As expected, since the gastrocnemius and biceps femoris muscles in this study were collected 24 h after the swimming until exhaustion test, no difference in the PGC-1α mRNA and protein levels of the Cy3G-untreated mice (“rest period”) was observed. However, even though the muscles samples were collected during the “rest period”, an increase in PGC-1α mRNA and protein expression was observed in Cy3G-treated group (Fig. 2). Furthermore, Cy3G also upregulated *LDH B* and *MCT1* expression (Fig. 2) suggesting that the observed increase in lactate metabolism through PGC-1α upregulation was due to Cy3G. Interaction of PGC-1α with estrogen-related receptor α (ERRα) induces lactate metabolism and angiogenesis^21^,^37^. As shown in Fig. 2, the expression of *VEGFα* was increased by Cy3G-induced PGC-1α upregulation. Muscle vascularization by VEGFα increases muscle blood supply and oxygen availability thus increasing exercise time and endurance^38^. This result suggests that the increase in *VEGFα* expression in muscle by Cy3G enhanced the mice’s performance during exercise.

MCT1 is involved lactate and ketone bodies (β-hydroxybutyrate, acetoacetate, and acetone) transport^39^ and an elevation of PGC-1α level in skeletal muscle increases MCT1 expression and plays a central role in ketone body homeostasis^33^. The increase in *MCT1* expression due to Cy3G, therefore, is associated with the observed decreased total ketone body levels in the serum (Table 1). Free fatty acids (FFAs) are metabolised via β-oxidation in the skeletal muscle^40^ and overexpression of PGC-1α activates the FFA oxidative capacity of skeletal muscle cells by increasing the expression of genes that are involved in lipid metabolism one of which is the carnitine palmitoyltransferase 1β (*CPT-1β*). CPT-1β, present in the outer membrane of mitochondria, is the rate-limiting enzyme of mitochondrial fatty acid uptake^41^,^42^. Cy3G caused a reduction in the non-esterified fatty acid levels in the serum, which is expected following PGC-1α upregulation (Table 1 and Fig. 2). These results indicate that the enhanced FFA metabolism was caused by Cy3G-induced PGC-1α upregulation. Peroxisome proliferator-activated receptor-δ (PPARδ), which is known as a regulator of lipid metabolism, is the most abundantly expressed of the PPAR family in skeletal muscle^43^. PGC-1α interacts with PPARδ and regulates lipid metabolism by increasing expression of the target genes such as *CPT-1β* and *UCP-3*^43^. Cyanidin, which is an aglycone of Cy3G, promotes the expression of PPARs and their target genes and acts as a ligand of the PPAR family, including PPARδ^44^. In this study, the expression of PGC-1α and PPARδ were upregulated by Cy3G (Fig. 2) and, the activation of FFA metabolism by Cy3G is most likely synergistically regulated by PGC-1α and PPARδ.

Mitochondrial number and function increase in skeletal muscle is an important adaptation to exercise^2^,^6^. Mitochondrial content of skeletal muscle cells was increased in response to Cy3G-induced *PGC-1α* upregulation (Fig. 4). PGC-1α interacts with ERRα to regulate the transcription of genes related to mitochondrial biogenesis, such as TFAM, and lactate metabolism^23^,^28^. *PGC-1α* upregulation by Cy3G, therefore, promoted mitochondrial biogenesis through interaction with ERRα. Intracellular nitric oxide (NO) regulates mitochondrial biogenesis and partly interacts with CaMKK and AMPK^45^,^46^. Xu JW *et al*.^47^ reported that Cy3G can increase intracellular NO by activating NO synthase in vascular endothelial cells while cAMP and PDE inhibitors regulates NO production^48^,^49^. In the current study, we report that intracellular cAMP levels were increased in response to Cy3G treatment (Fig. 5D). Therefore, the intracellular NO production could be partly involved in Cy3G-induced mitochondrial biogenesis.

The AMPK pathway promotes mitochondrial biogenesis by increasing the expression of PGC-1α^29^. CaMKK is activated when the intracellular Ca^2+^ level is increased, which then phosphorylates AMPK^50^. CaMKK signalling pathway is involved in the stable induction of PGC-1α by auto feedback regulation^51^. Here, Cy3G increased *PGC-1α* expression by elevating intracellular Ca^2+^ that in turn activated the CaMKK-AMPK pathway (Fig. 5). In a recent study done by Guo H *et al*.^52^, Cy3G was found to activate CaMKKβ–AMPK pathway by increasing intracellular Ca^2+^ levels in HepG2 hepatocytes. Furthermore, Cy3G caused the elevation in intracellular Ca^2+^ and cAMP levels in C2C12 myotubes (Fig. 5C and D). Elevation of cAMP levels was detected 15 min after Cy3G treatment but did not change the Ca^2+^ levels at this time. However, after 15 more min or 30 mins after treatment, an increase in Ca^2+^ level was detected. Epac1 induces the release of Ca^2+^ from the endoplasmic reticulum/sarcoplasmic reticulum, which is cAMP-regulated guanine nucleotide exchange factor^32^. We suggest that the elevation of intracellular cAMP levels by Cy3G was the underlying cause for the increased release of intracellular Ca^2+^. Phosphodiesterase (PDE), is an enzyme that hydrolyses cAMP to 5′AMP and regulates intracellular cAMP levels^53^. For the cAMP assay, PDE inhibitors were used to prevent cAMP hydrolysis and results showed that in the presence of PDE inhibitors, Cy3G increased the intracellular cAMP levels. But this effect was abrogated by the removed of PDE inhibitors (Fig. 5D). Dallas C *et al*.^54^ used an *in vitro* cell-free assay and reported that Cy3G has an inhibitory effect on PDE enzymatic activity. Therefore, we suggest that the increased intracellular cAMP level was due to the Cy3G-inhibited PDE activity.

**Figure 6:**
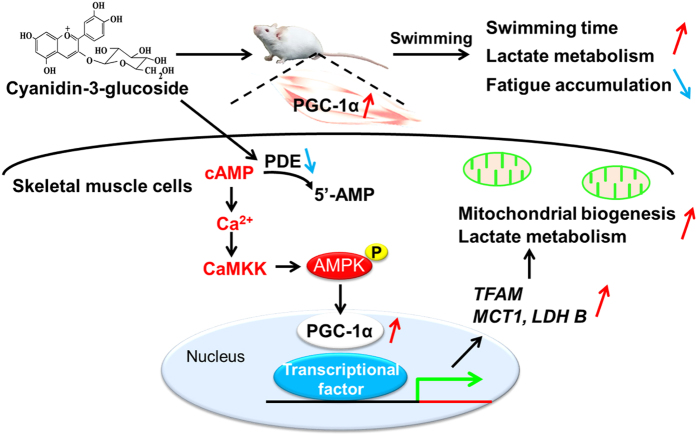
Skeletal muscle PGC-1α upregulation by Cyanidin-3-glucoside (Cy3G) enhances exercise performance. Increase of lactate metabolism in response to Cy3G-induced PGC-1α upregulation enhanced exercise performance and reduced fatigue. This PGC-1α upregulation is modulated by CaMKK–AMPK pathway via the elevation of intracellular cAMP levels.

In summary, we found that the increase of lactate metabolism in response to Cy3G-induced PGC-1α upregulation enhanced exercise performance in mice. The mitochondrial content of the muscle cells was increased by Cy3G-induced *PGC-1α* upregulation via the CaMKK–AMPK pathway that was influenced by the elevation of intracellular cAMP levels (Fig. 6). Here, we have demonstrated the regulatory effect of Cy3G on exercise performance and metabolic activity in skeletal muscle and suggest that nutraceuticals can increase PGC-1α expression which consequently enhance exercise performance.

## Materials and Methods

### Chemicals

Cy3G was purchased from Tokiwa Phytochemical Co. Ltd. (Chiba, Japan). ISOGEN was purchased from Nippongene (Tokyo, Japan). Rhodamine 123 and sodium dodecyl sulphate (SDS) were purchased from Wako (Tokyo, Japan). DMEM, 4-(2-hydroxyethyl)-1-piperazineethanesulfonic acid (HEPES), Triton X-100, RIPA buffer, protease inhibitor cocktail, β-Actin antibody, L-lactic acid, 3-isobutyl-1-methylxanthine (IBMX) and Ro 20-1724 were purchased from Sigma (MO, USA). Fetal bovine serum, house serum, Hanks’ balanced salt solution (HBSS) and DMEM (no glucose) were purchased from Gibco (NY, USA). MTT was purchased from Dojindo (Kumamoto, Japan). STO-609 was purchased from Focus Biomolecules (PA, USA). PGC-1α (3G6), AMPKα and phospho-AMPKα (Thr172) antibodies were purchased from Cell Signaling Technology (Hertfordshire, UK). Glyceraldehyde 3-phosphate dehydrogenase (GAPDH) antibody (6C5) was purchased from Santa Cruz Biotechnology (CA, USA).

### Animal experiments

Five week-old male ICR mice were obtained from Charles River Laboratories (Kanagawa, Japan) and maintained under a 12 h light/dark cycle and had free access to water and a normal diet (MF, Oriental Yeast Co., Ltd., Japan). After a week of acclimatization to laboratory condition, the mice were randomly divided into three groups based on their body weight, with each group having the same average weight: Group 1 (no exercise group) was orally administered with water without swimming exercise (n = 6). Groups 2 and 3 performed swimming exercise (n = 7 per group). Group 2 (control group) was orally administered with water. Group 3 (Cy3G group) was orally administered 1 mg/kg of Cy3G in water. Animals were orally administrated with 100 μl sample solution using animal feeding needles (Group 1 and 2: D.W. or Group 3: 1 mg/kg Cy3G dissolved in water). Oral administration was performed every day, 1 h before the swimming exercise and at the same time on the days when exercise was not performed. Body weight was measured daily while food intake was measured weekly. The exercise protocol was adapted from a study by Takeda K *et al*. with some modifications^22^. Swimming tests were carried out under non-fasted state to avoid energy depletion before performing the swimming exercise. Mice were trained to perform the swimming exercise for 10 min with a load corresponding to 5% of their body weight attached to their tails in a tank (30 × 30 × 40 cm) filled with water to a depth of 25 cm, kept at 30 ± 1 °C. The swimming exercise was performed every other day for 14 days and on day 15, swimming-until-exhaustion test was carried out. The mice were made to swim to exhaustion with a load corresponding to 10% of their body weight. Each mouse was considered to reach exhaustion when it failed to raise its face to the water surface within 5 s. Blood lactate levels were measured before and after exercise (0 and 60 min) using Lactate Pro 2 (Arkrey, Japan). Blood glucose levels were measured after exercise (0 min) using Glucose pilot (Iwai Chemicals Company, Japan). Blood samples for lactate and glucose determination were collected from the tail vein. The mice were subjected to 16 h fasting before tissues (liver, gastrocnemius and biceps femoris) and blood collection. Collected tissues were washed with PBS before freezing in liquid nitrogen. Total protein was isolated from tissue samples (10 mg) using RIPA buffer containing a protease inhibitor cocktail according to the manufacturer’s instructions. Quantification of the protein content of the tissue samples was done using a 2D Quant kit (GE Healthcare, Sweden). The serum was collected by centrifugation at 3000 ×  *g* for 10 min and biochemical parameters of serum were measured by Oriental Yeast Co., Ltd (Japan). All the animal experiments complied with the guidelines of the University of Tsukuba’s Regulation of Animal Experiments and were approved by the International Animal Care and Use Committee of the University of Tsukuba.

### Cell culture and differentiation

The mouse C2C12 myoblasts (ATCC, USA) were cultured in DMEM supplemented with 10% foetal bovine serum and 1% penicillin (5000 μg/ml)–streptomycin (5000 IU/ml) (Lonza, Tokyo, Japan). Normal human skeletal muscle myoblasts (Lonza) were cultured in a SkGM-2 Bullet Kit (Lonza) at 37 °C in a humidified atmosphere of 5% CO_2_. For differentiation into C2C12 myotubes, C2C12 myoblasts were cultured to confluence and then transferred to DMEM containing 2% horse serum and cultured for 5 days. The growth medium was changed every other day. For differentiation into HSMM, myoblasts were cultured to 60–70% confluence and then transferred to DMEM:F-12 (Lonza) containing 2% horse serum for 5 days. The growth medium was changed every other day.

### Real-time PCR analysis

Total RNA was isolated from tissue samples (50 mg), C2C12 myotubes and HSMM using ISOGEN. Total RNA isolation and TaqMan real-time PCR amplification reactions were performed as previously described^14^. For the quantification of gene expression in muscle tissues and C2C12 myotubes, the following specific TaqMan probes purchased from Applied Biosystems (CA, USA) were used: *β-actin* (Mm00607939_s1), *PGC-1α* (Mm01208835_m1), *PPARδ* (Mm00803184_m1), *TFAM* (Mm00447485_m1), *LDHa* (Mm01612132_g1), *LDHb* (Mm01267402_m1), *MCT1* (Mm01306379_m1), *CPT-1β* (Mm00487191_g1), *VEGFα* (Mm00437306_m1), *IGF1* (Mm00439560_m1), and *UCP-3* (Mm00494077_m1). *β-actin* (Hs01060655_g1), *PGC-1α* (Hs01016719_m1), *TFAM* (Hs00273327_s1), *UCP-3* (Hs01106052_m1), and *CPT-1β* (Hs03046298_s1) were used for HSMM. The mRNA expression levels of each gene was normalised using *β-actin* as an internal control.

### Western blotting

Total protein was isolated from tissue samples (10 mg) and C2C12 myotubes using RIPA buffer containing a protease inhibitor cocktail according to the manufacturer’s instructions. Protein samples (15 μg) were separated using 10% SDS-PAGE and transferred to a PVDF membrane (Merck Millipore, USA). Membranes were blocked using Odyssey blocking buffer (LI-COR, Inc., NE, USA) for 1 hour, and incubated with the following antibodies: PGC-1α (1:1000 dilution), AMPKα (1:1000 dilution), Phospho-AMPKα (Thr172) (1:1000 dilution), GAPDH (1:200 dilution) and β-Actin (1:5000 dilution), at 4 °C overnight, then incubated with secondary antibodies: IRDye 800CW donkey anti-rabbit IgG (1:10000 dilution) or IRDye 680LT goat anti-mouse (1: 20000 dilution) (LI-COR, Inc., NE, USA) at room temperature for 30 min. The signal was detected using the Odyssey Fc Imaging System (LI-COR, Inc., NE, USA).

### MTT assay

After treatment, C2C12 myotubes were incubated with MTT solution (5 mg/ml) for 3 h until formazan crystals were formed which were then dissolved in 10% SDS and incubated further for 16 h. Absorbance at 570 nm was then measured using a Powerscan HT plate reader (Dainippon Sumitomo Pharma Co, Ltd., Japan). The values were normalised to the value of the growth medium (blank).

### Measurement of intracellular ATP levels

The intracellular ATP levels in C2C12 myotubes were measured using Cellno ATP assay reagent (TOYO Ink, Japan) according to the manufacturer’s instructions. Briefly, after treatment, ATP assay reagents were added and incubated for 10 min at 25 °C. Luminescence was then measured using a Powerscan HT plate reader.

### Measurement of mitochondrial content using Rhodamine 123

Mitochondria content in C2C12 myotubes and HSMM was measured using a fluorescent dye, rhodamine 123^55^. After treatment, myotubes were incubated with rhodamine 123 (10 μg/ml) in 10 mM HEPES-HBSS buffer (pH 7.4) for 20 min at 37 °C. To quantify the rhodamine 123 content, myotubes were lysed using 1% Triton X-100 and the fluorescence intensity (excitation/emission 485/528 nm) was measured using a Powerscan HT plate reader.

### siRNA knockdown of *PGC-1α*

Differentiated C2C12 myotubes were transfected with *PGC-1α* siRNA (siRNA ID: s72017, Ambion, CA, USA) or *Silencer*^®^ Select Negative Control No. 1 siRNA (Ambion, CA, USA) for 48 h using Lipofectamine 3000 (Life Technologies, Japan) according to the manufacturer’s instructions. *Silencer*^®^ Select Negative Control No. 1 siRNA was used as negative control.

### Measurement of intracellular Ca^2+^ levels

Intracellular Ca^2+^ levels of C2C12 myotubes were measured using a Calcium Kit II-Fluo 4 (Dojindo, Japan) according to the manufacturer’s instructions. Briefly, differentiated C2C12 myotubes in black clear-bottom 96 well plates (Corning, NY, USA) were pre-incubated with 100 μl per well of loading buffer (5% Pluronic F-127, 250-mmol/l Probenecid and 1-μg/μl Fluo 4 AM in Hanks’–HEPES Buffer) for 30 min and subsequently treated with Cy3G for 15–90 min. Fluorescence intensity (excitation/emission 485/528 nm) was then measured using a Powerscan HT plate reader.

### Measurement of intracellular cAMP levels

The intracellular cAMP levels in C2C12 myotubes were measured using the cAMP-Glo^TM^ MAX assay (Promega, USA) according to the manufacturer’s instructions. Briefly, differentiated C2C12 myotubes in white, clear-bottom tissue culture 96 well plates (Corning, NY, USA) were cultured in serum-free DMEM containing 20 mM MgCl_2_ with or without PDE inhibitors (500 μM IBMX and 100 μM Ro20-1724) and with or without Cy3G for 30 min. Luminescence was then measured using a Powerscan HT plate reader. In this assay, PDE inhibitors were used to prevent cAMP hydrolysis during the assay.

### Statistical analysis

All results are expressed as the mean ± standard deviation, and statistical evaluation was performed using the Student’s t-test when two value sets were compared. Multiple comparisons were carried out using one way analysis of variance (ANOVA) followed by Tukey’s multiple comparison test using SPSS (IBM Statistics for Windows, version 22.0. IBM Corp, Armonk, NY). *P* ≤ 0.05 was considered to be statistically significant.

## Additional Information

**How to cite this article:** Matsukawa, T. *et al*. Upregulation of skeletal muscle PGC-1α through the elevation of cyclic AMP levels by Cyanidin-3-glucoside enhances exercise performance. *Sci. Rep.* **7**, 44799; doi: 10.1038/srep44799 (2017).

**Publisher's note:** Springer Nature remains neutral with regard to jurisdictional claims in published maps and institutional affiliations.

## Supplementary Material

Supplementary Information

## Figures and Tables

**Table 1:** The level of serum biochemical parameters in mice.

	No exercise	Swimming	
		Control	Cy3G
Urea nitrogen (mg/dL)	32.3 ± 0.9	28 ± 3.5	22 ± 1.8^**,#^
Creatinine (mg/dL)	0.15 ± 0.01	0.20 ± 0.05	0.12 ± 0.02^#^
Total ketone bodies (μM)	593.3 ± 53.3	1282.0 ± 210.4^**^	847.8 ± 143.5^*,##^
AST (IU/L)	1690.0 ± 258.9	2082.5 ± 334.3	2075.0 ± 388.9
ALT (IU/L)	226.3 ± 3.5	226.8 ± 40.4	160.2 ± 103.0
ALP (IU/L)	345.0 ± 17.9	433.7 ± 37.6**	347.5 ± 40.9^#^
NEFA (μEq/L)	1125.8 ± 140.9	1298.2 ± 129.8	1080.0 ± 122.6^#^

Values are expressed as the mean ± standard deviation. *P ≤ 0.05 and **P ≤ 0.01 indicate a significant difference from the no exercise group. ^#^*P* ≤ 0.05 indicates a significant difference from the control group. AST: aspartate transaminase, ALT: alanine transaminase, ALP: alkaline phosphatase, NEFA: non-esterified fatty acid.

**Table 2:** Body weight, food intake and the weight of liver, gastrocnemius and biceps femoris in mice.

	No exercise	Swimming	
		Control	Cy3G
Initial body weight (g)	28.7 ± 1.3	28.2 ± 1.4	28.5 ± 0.8
Final body weight (g)	30.7 ± 1.2	31.6 ± 2.0	32.5 ± 1.7
Food intake (g/day)	3.67 ± 0.12	3.67 ± 0.22	3.79 ± 0.16
Liver (g)	1.19 ± 0.04	1.18 ± 0.04	1.29 ± 0.04^**,##^
Gastrocnemius (g)	0.22 ± 0.03	0.23 ± 0.02	0.29 ± 0.04^*,#^
Biceps femoris (g)	0.26 ± 0.03	0.34 ± 0.0^**^	0.56 ± 0.02^**,##^

Values are expressed as the mean ± standard deviation. *P ≤ 0.05 and **P ≤ 0.01 indicate a significant difference from the no exercise group. ^#^*P* ≤ 0.05 and ^##^*P* ≤ 0.01 indicate a significant difference from the control group.
