# Supplementary material for: Upregulation of skeletal muscle PGC-1α through the elevation of cyclic AMP levels by Cyanidin-3-glucoside enhances exercise performance
Source: Sci Rep. 2017 Mar 20;7:44799. doi: 10.1038/srep44799 (PMC5357948; doi:10.1038/srep44799)
Supplement: Supplementary Information [file srep44799-s1.pdf]

Supplementary information

Upregulation of skeletal muscle PGC-1α through the elevation of cyclic AMP levels by Cyanidin-3-glucoside enhances exercise performance

Toshiya Matsukawa[1], Hideko Motojima[2], Yuki Sato[1], Shinya Takahashi[2,3], Myra O. Villareal[2,3] and Hiroko Isoda[2,3*]

[1]Graduate School of Life and Environmental Sciences, University of Tsukuba, Tsukuba City, Ibaraki 305-8572, Japan

[2]Alliance for Research on North Africa (ARENA), University of Tsukuba, Tsukuba City, Ibaraki 305-8572, Japan

[3]Faculty of Life and Environmental Sciences, University of Tsukuba, Tsukuba City, Ibaraki 305-8572, Japan

Supplementary Table 1. Protein content of liver, gastrocnemius and biceps femoris in mice

		Swimming	
	No exercise	Control	Cy3G
Liver (μg/mg)	85.8 ± 16.4	84.2 ± 7.1	90.8 ± 17.9
Gastrocnemius (μg/mg)	92.3 ± 3.76	91.2 ± 1.88	96.9 ± 1.52[##]
Biceps femoris (μg/mg)	94.7 ± 7.26	94.2 ± 6.54	96.0 ± 1.30

Protein content (μg)/ tissue weight (mg) is expressed as the mean ± standard deviation.

[##] $P < 0.01$ indicate a significant difference from the control group.

Supplementary Table 2. The ratio of tissue weight to body weight (mg/g)

		Swimming	
	No exercise	Control	Cy3G
Liver wt. /Body wt.	39.03 ± 2.12	38.24 ± 0.52	39.04 ± 1.32
Gastrocnemius wt./Body wt.	8.04 ± 0.47	7.66 ± 0.69	10.42 ± 1.01$^{**,\ \#\#}$
Biceps femoris wt./Body wt.	8.95 ± 1.30	10.75 ± 0.96*	16.85 ± 0.74$^{**,\#\#}$

The ratio of tissue weight to body weight (mg/g) is expressed as the mean ± standard deviation.

* $P < 0.05$ and ** $P < 0.01$ indicate a significant difference from the no exercise group.

$P < 0.01$ indicate a significant difference from the control group.

Supplementary Figure 1

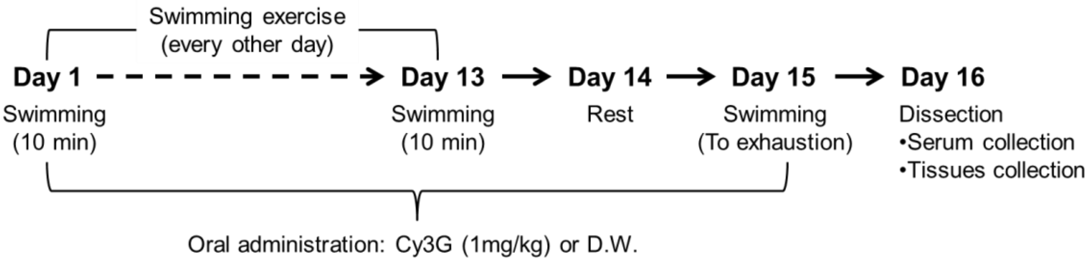

Figure S1. Experimental schedule of animal experiments

Mice were trained to perform a swimming exercise and performed the exercise every other day for 14 days, and an exhaustion swimming test was carried out on day 15. After 24 h recovery (Day 16), serum and tissues were collected.
